# Spinal cord NR1 serine phosphorylation and NR2B subunit suppression following peripheral inflammation

**DOI:** 10.1186/1744-8069-1-25

**Published:** 2005-09-02

**Authors:** Robert M Caudle, Federico M Perez, Arseima Y Del Valle-Pinero, Michael J Iadarola

**Affiliations:** 1 Department of Oral and Maxillofacial Surgery and Diagnostic Sciences, University of Florida College of Dentistry, Gainesville, FL 32610, USA; 2 Department of Neuroscience, University of Florida College of Medicine, McKnight Brain Institute, Gainesville, FL 32610, USA; 3 Pain and Neurosensory Mechanisms Branch, National Institutes of Dental and Craniofacial Research, National Institutes of Health, Bethesda, MD 20892, USA

## Abstract

**Background:**

Spinal cord N-methyl-D-aspartate (NMDA) receptors are intimately involved in the development and maintenance of central sensitization. However, the mechanisms mediating the altered function of the NMDA receptors are not well understood. In this study the role of phosphorylation of NR1 splice variants and NR2 subunits was examined following hind paw inflammation in rats. We further examined the level of expression of these proteins following the injury.

**Results:**

Lumbar spinal cord NR1 subunits were found to be phosphorylated on serine residues within two hours of the induction of hind paw inflammation with carrageenan. The enhanced NR1 serine phosphorylation reversed within six hours. No phosphorylation on NR1 threonine or tyrosine residues was observed. Likewise, no NR2 subunit phosphorylation was observed on serine, threonine or tyrosine residues. An analysis of NR1 and NR2 protein expression demonstrated no change in the levels of NR1 splice variants or NR2A following the inflammation. However, spinal cord NR2B expression was depressed by the hind paw inflammation. The expression of NR2B remained depressed for more than one week following initiation of the inflammation.

**Conclusion:**

These data suggest that NR1 serine phosphorylation leads to an initial increase in NMDA receptor activity in the spinal cord following peripheral injury. The suppression of NR2B expression suggests compensation for the enhanced nociceptive activity. These data indicate that spinal cord NMDA receptors are highly dynamic in the development, maintenance and recovery from central sensitization following an injury. Thus, chronic pain therapies targeted to NMDA receptors should be designed for the exact configuration of NMDA receptor subunits and post-translational modifications present during specific stages of the disease.

## Background

Central sensitization is a form of plasticity in the spinal cord that alters the input/output relationship of the neuronal pain processing circuitry. Central sensitization is symptomatically expressed as allodynia, pain to normally non-painful stimuli, and hyperalgesia, an enhanced sensation of pain to typically painful stimuli. When an individual is injured central sensitization encourages the protection of the injured area by enhancing the pain experience. The individual is then motivated to guard the damaged tissue until it is healed. As a rule, central sensitization will be reversed as the injury heals. However, on occasion it fails to resolve and becomes the patient's primary disease. This disease is referred to as chronic pain. Thus, the molecular processes that induce and reverse central sensitization are important to understanding, preventing and treating chronic pain.

Recent work on pain processing has highlighted the central role of N-methyl-D-aspartate (NMDA) receptors in central sensitization. NMDA receptors were found to play a major role in hyperalgesia, allodynia, and expanded receptive fields when central sensitization had been induced by peripheral injury [1-5]. These findings using NMDA receptor antagonists indicated that NMDA receptors initiated events that lead to neuronal plasticity in the spinal cord and that the NMDA receptors themselves participated in the maintenance of central sensitization. Central sensitization is the result of an increase in intracellular calcium, which enhances synaptic inputs from primary nociceptors. NMDA receptors conduct much of this calcium from the extracellular space through their ionophore. The net effect of the increased calcium is an increased number of effective synapses on dorsal horn neurons and enhanced neuronal excitability [1,6,7].

Central sensitization, it must be noted, is distinct from the frequently studied phenomenon of windup, which is rapidly reversed when the peripheral stimulus ceases. Windup is produced by the well documented voltage dependent magnesium block of the NMDA receptor's ion channel. The magnesium block enables the receptor to integrate nociceptive signals that arrive in the spinal cord via C-fibers. The net result of the integration is that the later stimuli in a series produces greater responses in dorsal horn neurons even when the stimuli are identical to the first event [8-10]. Windup does not lead to a prolonged enhancement of dorsal horn neuronal excitability like central sensitization, but may induce central sensitization by increasing intracellular calcium levels. Thus, although NMDA receptors are involved in both central sensitization and windup their role in the two processes is distinct [10].

Recently, Zou and colleagues examined the role of NMDA receptor subunit phosphorylation in the development of central sensitization [11]. These investigators found that capsaicin injection into the hind paw of rats resulted in an ipsilateral accumulation of phosphorylated NR1 subunits in spinothalamic tract neurons. Zou detected the phosphorylation using an antibody that was selective for phosphorylated serine 897 on the NR1 subunit. Phosphorylation of serine 897 on NR1 results in the accumulation of NMDA receptors in synapses [12]. Zou et al. further demonstrated that PKA mediated the phosphorylation of serine 897 and that the enhanced activity of spinothalamic tract neurons was sensitive to PKA inhibitors[13]. Similarly, Brenner and colleagues demonstrated that noxious heat applied to the hind paw of rats produces an increase in serine 896 phosphorylation of NR1 [14]. This phosphorylation was demonstrated to be mediated by activation of protein kinase C (PKC). Previously, it was demonstrated that PKC phosphorylation of serine 896 acts in concert with PKA phosphorylation of serine 897 to induce membrane insertion of NMDA receptors [15]. The findings from these two groups indicate that a relatively brief nociceptive stimulus in the periphery might induce the migration of new NMDA receptors to synapses between the primary and secondary nociceptive afferents. Presumably, the newly inserted NMDA receptors participate in enhancing synaptic activity and in inducing central sensitization. In contrast to the NR1 serine phosphorylation it was notable that these short duration stimuli did not induce any detectable change in NR1 protein expression.

In an excitotoxity induced spinal cord injury model, we examined NR1 phosphorylation and found that an increase in NR1 serine phosphorylation was associated with spontaneous behaviors that indicate the expression of hyperalgesia and allodynia [16]. We further found that, in contrast to the short term peripheral nociception models, NR1 protein was up regulated in the spinal cord following the excitotoxic injury. These data suggest that sustained injuries produce not only changes in NMDA receptor phosphorylation, but also changes in protein expression. The changes in NMDA receptor protein could lead to a more extended period of central sensitization than the readily reversed NMDA receptor phosphorylation.

In addition to NR1 subunit phosphorylation, it was recently demonstrated that NR2B subunits in the spinal cord were phosphorylated on tyrosine residues following either hind paw inflammation with Complete Freund's Adjuvant (CFA) or mustard oil [17]. The NR2B phosphorylation was blocked by inhibitors of Src type kinases. Interestingly, NR2A was reported not to be phosphorylated following the CFA injury. In addition, these investigators reported no change in the expression of NR2 protein in the spinal cord following the CFA treatment.

Overall, the NMDA receptor phosphorylation studies suggest that phosphorylation of spinal cord NR1 and NR2B subunits leads to enhanced NMDA receptor function and central sensitization. However, it is unclear if extended periods of central sensitization are maintained by NMDA receptor phosphorylation or if changes in NMDA receptor subunit protein expression contribute to the continuance of the sensitized state as was observed in the spinal cord injury model [16]. In the present study we examined phosphorylation on serine, threonine and tyrosine residues, as well as protein expression, in both NR1 and NR2 subunits following carrageenan induced hind paw inflammation. The goal was to determine if there is a transition from NMDA receptor subunit phosphorylation to changes in NMDA receptor subunit protein expression during an extended period of nociceptive input and the relationship of these changes to central sensitization.

## Results

### Characterization of NR1 Splice Variant Selective Antibodies

**Figure 1:**
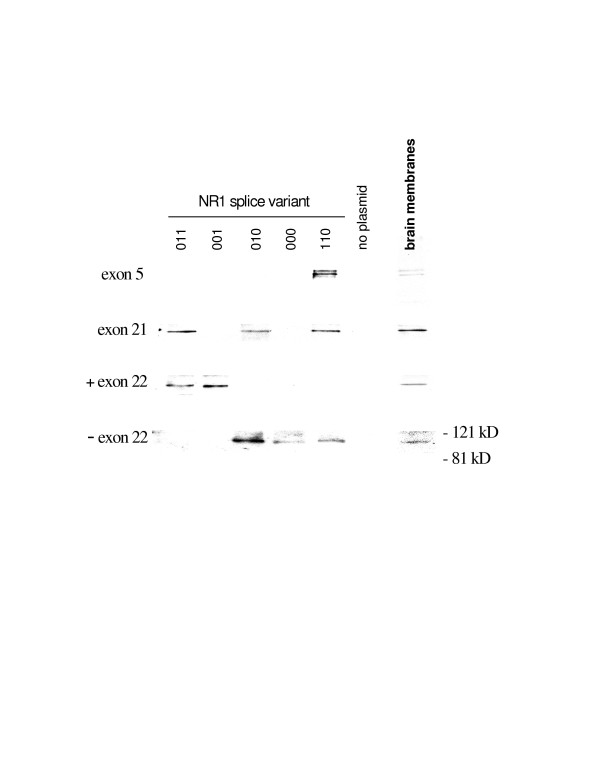
Selectivity of rabbit polyclonal antibodies raised against splice variants of the NR1 subunit of the NMDA receptor. Antibodies were raised against synthetic peptides and used for western blots. The peptide sequences used to raise the antibodies were SKKRNYENLDQLSYDNKRGPC, DRKSGRAEPDPKKKATFRAC, PRRAIEREEGQLQLC, and QYHPTDITGPLNLSDPS for exons 5 (N1), 21 (C1), 22 (C2) and 22 minus (C2') respectively. The antibodies were purified with affinity columns and tested against cloned splice variants of the NR1 subunit transfected into COS-7 cells. The three numbers at the top of the columns identify the splice variant clones. The first number indicates the presence (1) or absence (0) of N1, the second C1 and the third C2. The protein was compared to protein taken from non-transfected cells and rat brain homogenates.

Four antibodies were raised in rabbits against four regions of the NR1 subunit for identifying specific splice variants containing the N1, C1, C2 and C2' cassettes [18]. NR1 splice variant clones were transfected into COS-7 cells and the protein was harvested and run on western blots. The western blots were probed with the antibodies. As demonstrated in figure 1 the western blots demonstrated that the antibodies were highly selective for the appropriate NR1 splice variants.

### Time Course of Carrageenan Induced Enhancement of Nociception

**Figure 2:**
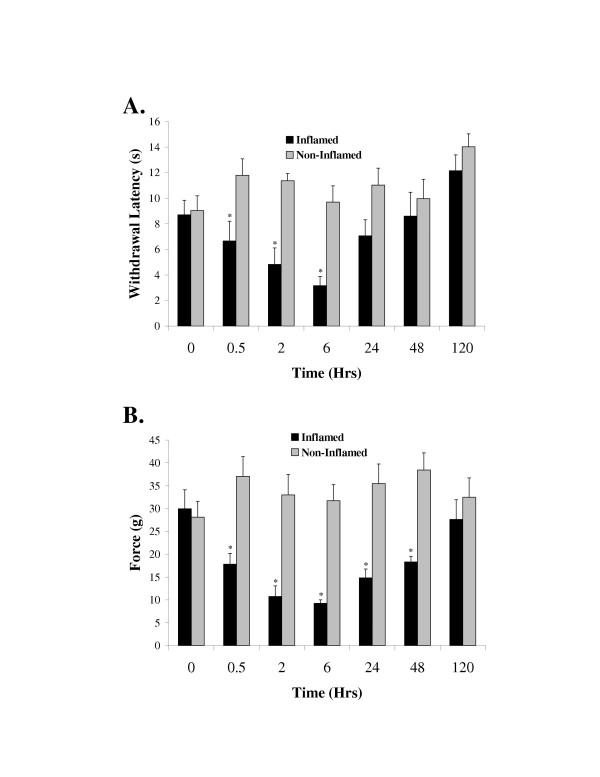
Time course of carrageenan induced inflammation on heat hyperalgesia and mechanical allodynia. **A**. Paw withdrawal latencies from a thermal stimulus before and following injection of 6 mg carrageenan into the plantar surface of the left hind paw. Data are means ± SEM (N = 8 rats). Asterisks indicate p < 0.05 ANOVA followed by Dunnett's test. **B**. Thresholds for paw withdrawal from a mechanical stimulus before and following the injection of 6 mg carrageenan into the plantar surface of the left hind paw. Data are means ± SEM (N = 8 rats). Asterisks indicate p < 0.05 ANOVA followed by Dunnett's test.

Sixteen rats received injections of 6 mg of carrageenan into the left hind paw and another sixteen rats received vehicle injections. Eight of the carrageenan rats and eight of the vehicle treated rats were then used for thermal nociceptive testing and the remaining animals were used for the mechanical nociception testing. The time course of the thermal testing demonstrated that the limb withdrawal latency was reduced by carrageenan injection within half an hour (Figure 2A). This reduced withdrawal latency reached a peak by 2 to 6 hours and recovery to baseline levels was achieved within 24 hours (ANOVA, Dunnett's test p < 0.05). Although there was a trend for greater thermal hyperalgesia at 6 hours when compared to the 2 hour time point this difference did not reach significance (paired ttest, p = 0.12). In contrast the non-injected paws demonstrated no significant changes in withdrawal latency. No change in paw withdrawal latency was observed in the vehicle injected animals (data not shown).

In the mechanical allodynia assay the carrageenan injected paws demonstrated significantly enhanced sensitivity to mechanical stimuli within half an hour. The enhanced sensitivity reached a peak within 6 hours. However, in contrast to the thermal assay the rats did not completely return to baseline levels for at least 2 days, but were fully recovered by 5 days (Figure 2B). The non-injected paws and vehicle treated animals (data not shown) demonstrated no significant change in mechanical sensitivity during the entire time course.

### NR1 Phosphorylation

To evaluate NR1 phosphorylation rats were injected with either vehicle or 6 mg of carrageenan into the left hind paw. The ipsilateral lumbar spinal cord tissue was harvested and NR1 subunits were precipitated with selective antibodies for the N1 or C1 cassettes [18] or an antibody that recognizes all 8 of the NR1 splice variants. The splice variant selective antibodies were raised in house (Figure 1). The protein was then run on western blots and probed with anti-phosphoserine, anti-phosphothreonine or anti-phosphotyrosine antibodies. A band at ~118 kd was identified by the anti-phospho antibodies, which was consistent with NR1 [14].

**Figure 3:**
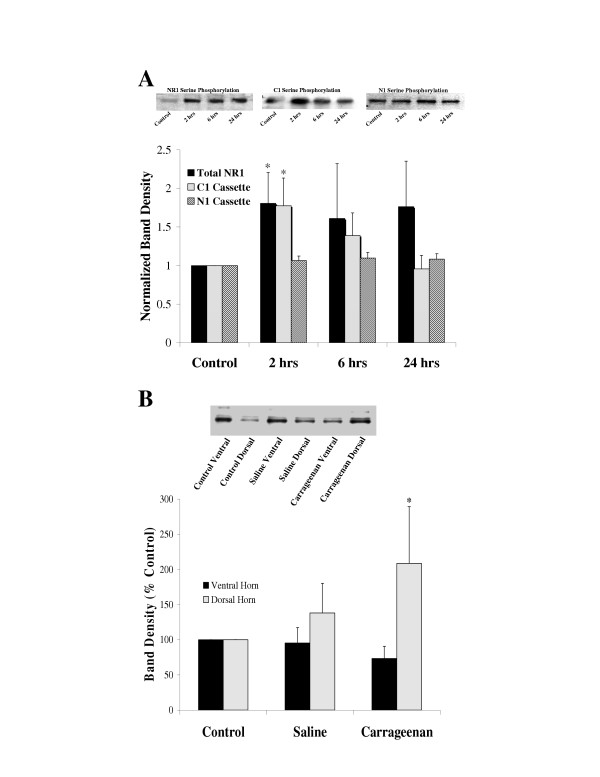
Serine phosphorylation of spinal cord NR1 subunits following carrageenan induced hind paw inflammation. **A**. Time course of serine phosphorylation on NR1 subunits immunoprecipitated by antibodies that recognized all NR1 subunits, N1 containing splice variants or C1 containing splice variants. The western blots at the top are representative experiments where the immunoprecipitated protein was probed with anti-phosphoserine antibodies. The bands migrated at approximately 118 kd. The graph summarizes the band density data from the western blots. Data are means ± SEM of the normalized band density data (NR1: N = 6 rats per time point, N1: N = 5 rats per time point, C1: N = 6 rats per time point). Asterisks indicate p < 0.05 ANOVA followed by Dunnett's test on the raw data. **B**. Comparison of serine phosphorylation on NR1 subunits from the dorsal and ventral spinal cord four hours following the injection of either saline or carrageenan (6 mg) into the left hind paws. At the top is a representative western blot demonstrating the immunoprecipitated NR1 after it was probed with anti-phosphoserine antibodies. The graph is a summary of the normalized band density data for control animals (N = 5), saline injected animals (N = 5) and carrageenan injected animals (N = 5). Data are means ± SEM. Asterisks indicate p < 0.05 ANOVA followed by Dunnett's test.

Carrageenan injected into the left hind paw induced a significant increase in serine phosphorylation of NR1 protein precipitated with the globally reactive NR1 antibody (N = 6 rats/time point) and with protein precipitated with the C1 cassette selective antibody (N = 8 rats/time point) within 2 hours of the carrageenan injection (ANOVA followed by Dunnett's test on raw data, p < 0.05) (Figure 3A). The phosphorylation of serine residues begins to resolve within 6 hours of the treatment. Interestingly, NR1 protein precipitated with N1 cassette selective antibodies did not demonstrate an increase in serine phosphorylation in response to carrageenan treatment (N = 8 rats per time point, ANOVA followed by Dunnett's test on raw data, p > 0.05).

To determine if the changes in phosphorylation were in the dorsal horn the isolated spinal cord segments of 8 carrageenan injected rats, 8 saline injected rats and 8 non-injected rats were further divided into dorsal and ventral halves and analyzed for serine phosphorylation. Two hours following the carrageenan injections the animals demonstrated an increase in serine phosphorylation in the dorsal horn, but not in the ventral horn (Figure 3B) (ANOVA followed by Dunnett's test on raw data, p < 0.05).

**Figure 4:**
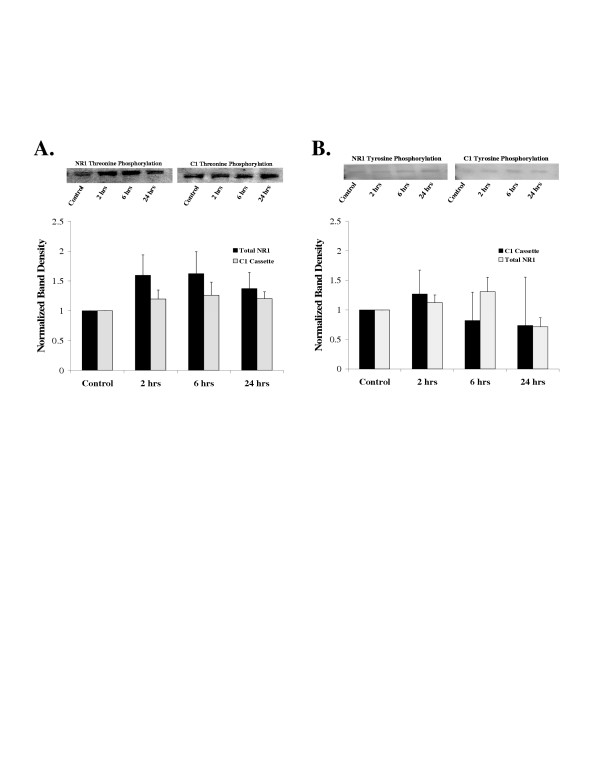
Phosphorylation of threonine and tyrosine residues on spinal cord NR1 subunits. No phosphorylation of NR1 threonine or tyrosine residues was found in response to peripheral inflammation. **A**. Lumbar spinal cord NR1 subunits were immunoprecipitated with an antibody that recognizes all NR1 splice variants or an antibody that recognizes splice variants with the C1 cassette before and after left hind paw inflammation with carrageenan. The protein was run on western blots and probed with an anti-phosphothreonine antibody. The western blots at the top are representative experiments and the graph at the bottom presents the mean ± SEM band density of all experiments (Total NR1; N = 8 rats per time point, C1 cassette; N = 9 rats per time point). ANOVA p > 0.05. The molecular weight of the bands on the western blots was ~118 kd. **B**. NR1 subunit protein was immunoprecipitated with an antibody that recognizes all NR1 splice variants or an antibody that recognizes splice variants with the C1 cassette, run on western blots and probed with an anti-phosphotyrosine antibody. The western blots at the top are representative experiments and the graph at the bottom presents the mean ± SEM band density of all experiments (Total NR1; N = 4 rats per time point, C1 cassette; N = 4 rats per time point). ANOVA p > 0.05.

Phosphothreonine and phosphotyrosine antibodies demonstrated no changes in phosphorylation of C1 precipitated NR1 (N = 9 rats/time point) or total NR1 protein (N = 8 rats/time point) (Figure 4A and 4B) (ANOVA followed by Dunnett's test on raw data, p < 0.05). The western blots probed with the anti-phosphotyrosine antibodies were extremely faint (see Figure 4B) indicating that NR1 is not phosphorylated on tyrosine residues to any significant degree.

### NR2 Phosphorylation

The NR2 subunits NR2A and NR2B were immunoprecipitated from rat spinal cord extracts as described in the methods section, run on western blots and probed with anti-phosphoserine, anti-phosphothreonine or anti-phosphotyrosine antibodies. The anti-phospho antibodies recognized proteins on the western blots with a molecular weight of approximately 180 kd, which is consistent with NR2A and NR2B [17].

**Figure 5:**
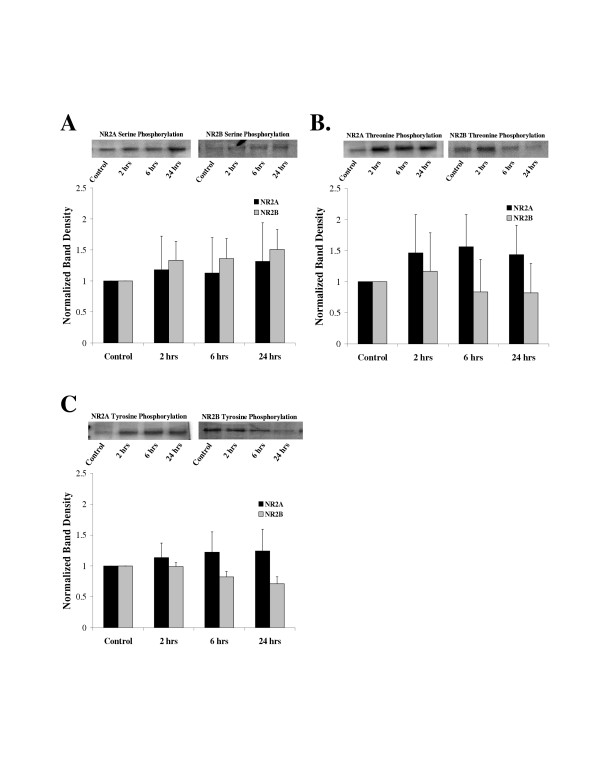
Phosphorylation of NR2A and NR2B subunits. NR2A and NR2B were immunoprecipitated from rat lumbar spinal before and after carrageenan induced inflammation of the left hind paw. The protein was run on western blots and probed with anti-phosphoserine, anti-phosphothreonine or anti-phosphotyrosine antibodies. Single bands were identified on the western blots that migrated at ~180 kd. **A**. Phosphoserine on NR2A and NR2B subunits. The western blots at the top demonstrate representative experiments and the graph below presents the mean ± SEM band density for all experiments (NR2A N = 4 rats per time point, NR2B N = 4 rats per time point). ANOVA p > 0.05. **B**. Phosphothreonine on NR2A and NR2B subunits. The western blots at the top demonstrate representative experiments and the graph below presents the mean ± SEM band density for all experiments (NR2A N = 4 rats per time point, NR2B N = 4 rats per time point). ANOVA p > 0.05. **C**. Phosphotyrosine on NR2A and NR2B subunits. The western blots at the top demonstrate representative experiments and the graph below presents the mean ± SEM band density for all experiments (NR2A N = 4 rats per time point, NR2B N = 4 rats per time point). ANOVA p > 0.05.

An examination of phosphorylation of NR2A (N = 5 rats/time point) and NR2B (N = 9 rats/time point) demonstrated that there was no significant change in phosphorylation on serine residues (Figure 5A) (ANOVA on raw data, p > 0.05) after carrageenan treatment. Similarly, no change in threonine or tyrosine phosphorylation was detected on either NR2A (N = 6 rats/time point) or NR2B (N = 5 rats/time point) (Figure 5B and 5C) (ANOVA on raw data, p > 0.05).

### NR1 Protein

**Figure 6:**
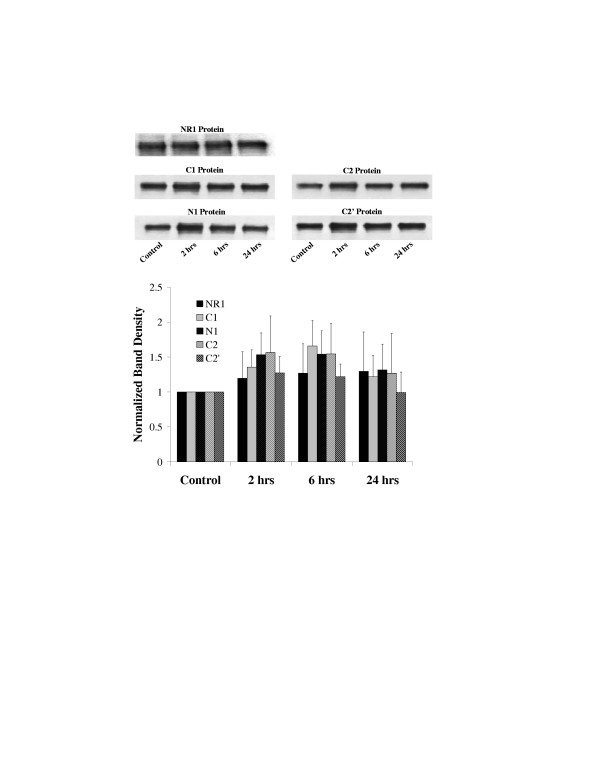
Peripheral inflammation does not influence NR1 protein expression in the lumbar spinal cord. Spinal cords were harvested before and after carrageenan injection into the left hind paw of rats, homogenized and run on western blots. The western blots were probed with antibodies that recognized all NR1 splice variants (N = 6 rats per time point), splice variants containing the N1 cassette (N = 8 rats per time point), splice variants with the C1 cassette (N = 8 rats per time point), splice variants with the C2 cassette (N = 4 rats per time point) and splice variants with the C2' cassette (N = 4 rats per time point). The western blots at the top are representative experiments and the graph a summary of all experiments. Bars are the mean ± SEM. ANOVA p > 0.05.

In western blots of spinal cord extracts the NR1 proteins were probed with antibodies selective for the N1 (N = 8 rats/time point), C1 (N = 8 rats/time point), C2 (N = 4 rats/time point) or C2' (N = 4 rats/time point) cassettes and all NR1 splice variants (N = 6 rats/time point). Extracts were prepared from animals without carrageenan injections, and 2, 6 and 24 hours following carrageenan injection into the hind paw. All of the antibodies recognized single bands on the westerns with a molecular weight of approximately 118 kd. Lane loading was monitored by probing for actin (data not shown). No change in spinal cord NR1 protein or splice variant expression was found following carrageenan inflammation (Figure 6) (ANOVA on the raw data, p > 0.05).

### NR2 Protein

**Figure 7:**
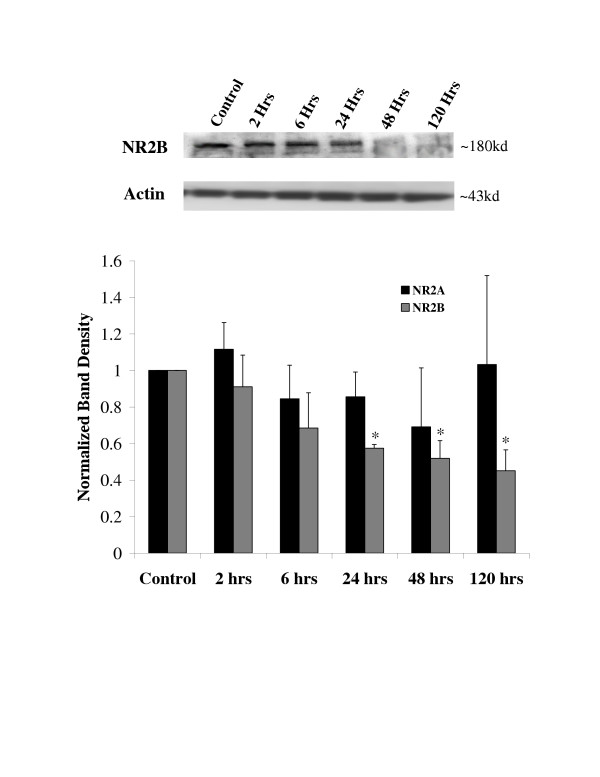
Expression of spinal cord NR2A and NR2B protein following peripheral inflammation. Rat lumbar spinal cords were harvested before and after carrageenan induced hind paw inflammation. The tissue was homogenized and run on western blots. The membranes were probed with antibodies that were selective for either NR2A (N = 4 rats per time point) or NR2B (N = 4 rats per time point). Single bands were identified at ~180 kd. The western blots are representative for NR2B and actin. Bars on the graph are means ± SEM of band density data normalized to the control bands. Asterisks indicate p < 0.05 ANOVA followed by Dunnett's test on the raw band densities.

To examine changes in NR2 protein spinal cord extracts were collected at 2, 6, 24, 48 and 120 hours following carrageenan injections and probed on western blots with antibodies to NR2A (N = 4 rats/time point) or NR2B (N = 4 rats/time point). Lane loading was monitored with antibodies to actin. The western blots for NR2A indicated no significant change in the expression of protein (ANOVA on raw data, p > 0.05) (Figure 7). However, there was a significant decrease in the expression of NR2B that was observed 24 hours following the carrageenan injection (ANOVA, Dunnett's test on raw data, p < 0.05). This decrease in NR2B lasted throughout the remainder of the week.

## Discussion

The involvement of spinal cord N-methyl-D-aspartate (NMDA) receptors in hyperalgesia and allodynia is well documented in the literature [2-5,19-21]. However, the mechanisms by which the receptors mediate the development and maintenance of allodynia and hyperalgesia in a chronic pain state are not well understood. Long lasting changes in the NMDA receptors either through post-translational modifications or expression of different subunits may lead to a chronic enhancement of nociception. In this study we used a relatively long lasting nociceptive stimulus to evaluate NR1 and NR2 subunit phosphorylation and protein expression. We found that spinal cord NR1 subunits possessing the C1 cassette are phosphorylated for a few hours following carrageenan injection (Figures 2 and 3). The NR1 phosphorylation was found only in the dorsal horn indicating that the phosphorylation was associated with nociception. Furthermore, the NR1 serine phosphorylation paralleled thermal hyperalgesia with recovery within 24 hours. The serine phosphorylation peaked at 2 hours following the carrageenan injections while the thermal hyperalgesia peaked in the range of 2 to 6 hours. This finding suggests that the NR1 serine phosphorylation may be a contributing factor to the thermal hyperalgesia.

The inflammation did not produce phosphorylation of serines on NR2 subunits or phosphorylation of threonine and tyrosine residues on either NR1 or NR2 subunits. The NR1 phosphorylation data are consistent with the shorter duration stimuli used by Zou et al. [11,13] and Brenner et al. [14], and with our previous spinal cord injury study [16]. However, we did not find that NR2B subunits were phosphorylated on tyrosine residues as was reported by Guo et al. [17]. The reason for this discrepancy is not known, but it could be due to the differences in inflammation protocols. We used carrageenan to inflame the hind paws while Guo et al. used Complete Freund's Adjuvant (CFA) to induce inflammation.

In a previous study, Prybylowski and colleagues demonstrated that NR1 subunits containing the C1 cassette in the spinal cord of rats represented only about 5 percent of the total NR1 protein [22], which suggests that C1 containing NR1 subunits may not play a significant role in the spinal cord. However, the group did not determine whether these NR1 subunits were localized to any specific area of the cord such as the dorsal horn or whether they were distributed throughout the lamina. It is possible that the C1 containing NR1 subunits are localized primarily in the superficial lamina, thus enhancing their abundance within neurons in the nociceptive pathways. From the work of Brenner et al. [14] and Zou et al. [13] it is clear that NR1 subunits are readily phosphorylated on serines 890, 896 and 897 by serine/threonine kinases during nociception, which is consistent with our findings. These serine residues reside in the C1 cassette indicating that NR1 subunits containing C1 are critical to nociceptive processing in the spinal cord. Interestingly, Prybylowski et al. also demonstrated that the majority of the NR1 subunits that contain N1 do not contain the C1 cassette [22]. This finding is consistent with our data demonstrating that when N1 is used as the precipitating epitope we do not find any injury induced phosphorylation of NR1 (Figure 3A) suggesting an absence of C1 in the precipitated protein. The sum of the data therefore suggests that NR1 subunits containing the C1 cassette and lacking the N1 cassette represent a dynamic pool of NMDA receptors that are rapidly recruited to the neuronal membrane following a nociceptive stimulus.

We further investigated the expression of NR1 splice variants, NR2A and NR2B protein. These data indicated no significant changes in NR1 splice variants or NR2A protein (Figures 6 and 7). However, NR2B expression was depressed by 24 hours following the carrageenan inflammation and did not recover during the 5 day period examined. The lack of change in NR1 subunits is consistent with the findings of Zou et al. [11,13], Brenner et al. [14] and Gaunitz et al. [23], but not with our spinal cord injury study [16]. These findings suggest that NR1 protein expression is not influenced by peripheral injuries, but is altered by central nervous system injuries. NR2B protein expression, in contrast, was suppressed by the peripheral inflammation. Guo et al. [17] did not find a change in NR2B protein following CFA induced peripheral inflammation and Gaunitz et al. [23] found no change in NR2B mRNA 6 hours following peripheral formalin injection. However, Gaunitz et al. did find a modest increase in NR2A and NR2C mRNA. From our findings we may conclude that the NR2A mRNA increases may not translate into increased levels of NR2A protein. We, however, did not examine NR2C protein.

**Figure 8:**
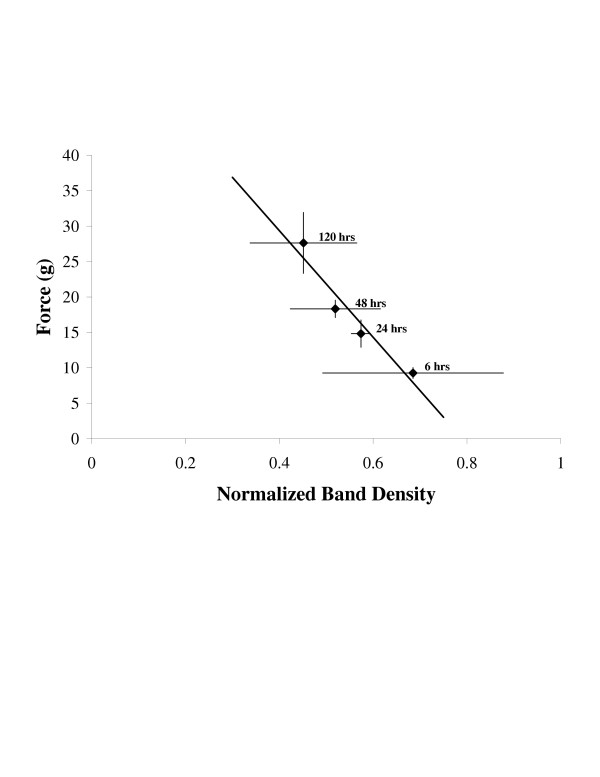
Correlation between NR2B protein and recovery of mechanical allodynia following carrageenan induced hind paw inflammation. Data from 6 hours to 120 hours from the NR2B western blots (figure 7) and the mechanical allodynia data (figure 2) were plotted against each other. Deming linear regression indicates r^2 ^= -0.93, p = 0.0363.

The suppression of NR2B following peripheral inflammation could have significant effects on nociception and the choice of treatments for chronic pain. NR2B subunits are located primarily in laminas I and II of the dorsal horn [24]. These subunits are involved in windup [25] and central sensitization [26] suggesting a major role for NR2B subunits in the function of NMDA receptors mediating nociception. Furthermore, recent work by Tan and colleagues [27] demonstrated that selective knockdown of NR2B in the dorsal horn using siRNA can suppress formalin induced nocifensive behaviors. Interestingly, our data demonstrate a strong negative correlation between the recovery period of mechanical allodynia and the level of NR2B protein expression (Figure 8, Deming linear regression, r^2 ^= -0.93, p = 0.0363). This might indicate that the suppression of NR2B is used to compensate for the enhanced nociceptive barrage. It is also possible; however, that NR2B is replaced by either NR2C or NR2D during a persistent nociceptive barrage. Thus, the NMDA receptors may still remain functional as the NR2B protein decreases. We have not yet examined NR2C or NR2D to confirm this hypothesis.

Several investigators have demonstrated that pharmacological agents that target NR2B subunits, such as Ifenprodil, can be used to control pain [28-31]. The loss of NR2B subunits in the spinal cord as the nociceptive stimulus progresses suggests that pharmacological agents targeting NR2B may be less effective in chronic pain than in acute pain. This idea is supported by the work of Nakazato and colleagues and De Vry et al. who recently demonstrated that intrathecally administered NR2B selective agents are ineffective at blocking mechanical allodynia in nerve constriction injury models [32,33]. These findings contrast with data that demonstrate NR2B selective antagonists suppress allodynia in acute nociception models [34] and that intrathecal siRNA knockdown of NR2B suppresses formalin induced nociceptive behaviors [27]. The efficacy of intrathecal NR2B antagonists in acute models and the lack of effect in chronic injury models support the concept that NR2B is down regulated in the spinal cord as the injury progresses.

The temporal association of thermal hyperalgesia with NR1 phosphorylation while recovery of mechanical allodynia is correlated with the suppression of NR2B suggests that the two behavioral phenomena have distinct mechanisms. Zou et al. and Brenner et al. have demonstrated that NR1 phosphorylation is mediated by the serine/threonine kinases protein kinase C (PKC) and protein kinase A (PKA) [11,13,14]. Their findings suggest that phosphorylation of NR1 induces the migration of NMDA receptors from the endoplasmic reticulum to synapses leading to enhanced transmission of nociceptive information [14,35,36]. It is possible that the phosphorylated receptors remain in the synapses and remain functional following dephosphorylation. The dephosphorylation of the receptors may reduce stimulation of dorsal horn neurons produced by C-fibers transmitting thermal information. In contrast, the enhanced number of NMDA receptors remaining in synapses may still conduct a significant amount of information from A-delta fibers that transmit mechanical information. Only as the number of NR2B subunits is reduced do the synapses return toward normal function. Presumably, the loss of NR2B subunits would prevent the remaining NR1 subunits from forming functional receptors in much the same way as siRNA to NR2B was found to suppress nociceptive behaviors by selectively reducing the amount of NR2B protein [27]. Thus, thermal hyperalgesia may be mediated by the enhanced function of phosphorylated NMDA receptors, while mechanical allodynia may simply require more NMDA receptors in the synapse.

Alternatively, the phosphorylated NR1 subunits may be inserted into C-fibers synapses while the NR2B subunits are located primarily in synapses associated with A-delta fibers. Thus, in the sensitized neurons suppression of the NR1 phosphorylation results in reduction of thermal hyperalgesia and a decrease in NR2B expression produces an inhibition of mechanical allodynia. These hypotheses are highly speculative at this time as the differences observed between the two forms of stimuli may actually be mediated by changes in the periphery rather than centrally. However, it is intriguing to consider that thermal hyperalgesia might be controlled via kinase inhibitors, whereas established mechanical allodynia may require selective NMDA receptor antagonists or suppression of NMDA receptor gene expression to be controlled.

In summary, our data indicate that peripheral inflammation with carrageenan results in a transient increase in serine phosphorylation of spinal cord dorsal horn NR1 subunits and a long lasting decrease in the expression of NR2B. These data indicate that NMDA receptors in the spinal cord are highly dynamic and may represent a moving target for pharmacological control of chronic pain.

## Methods

Male Sprague Dawley rats (200–300 g, Harlan Sprague Dawley, Indianapolis, IN) were maintained on a 12 hour light/dark cycle and fed standard rodent chow and water ad libitum. All experiments were approved by the University of Florida Institutional Animal Care and Use Committee.

### Hind Paw Inflammation

Inflammation was induced in the left hind paw of the rats by subcutaneous injection of carrageenan (6 mg in 150 μl of saline) (Sigma, St. Louis, MO) into the plantar surface. Control animals received only saline (150 μl) injections. Animals used for behavioral tests were tested prior to the inflammation and at 0.5, 2, 6, 24, 48 and 120 hours following the injections. Rats used for western blots were euthanized (CO_2 _inhalation) and the spinal cord tissue was harvested at 2, 6 and 24 hours following the hind paw injections. For the analysis of NR2 proteins tissue was harvested at 2, 6, 24, 48 and 120 hours following the hind paw inflammation.

### Thermal Nociception Assay

Thermal nociception was measured using the method of Hargreaves et al. [37]. Briefly, the rats were placed on a clear plastic surface and allowed 15 minutes to accommodate to the enclosure. An infrared light was directed onto a hind paw's plantar surface approximately in the middle of the foot. The latency for the animal to remove its foot from the path of the light was used as the dependent measure for thermal sensitivity. The light intensity was initially adjusted to produce latencies of approximately 8 s and a cutoff time of 20 s was used to prevent injury to the animals. Both hind paws were tested 3 times and the average of the 3 tests was used as the paw withdrawal latency for that time point. A rest period of at least 2 minutes was observed before the animals were retested to prevent sensitization of the paws.

### Mechanical Allodynia Assay

An electronic Von Frey device (Ugo Basile, Italy) was used for analyzing mechanical sensitivity in the rats. The animals were placed on a wire screen and allowed 15 minutes to become accustomed to the device. A small steel rod (~0.5 mm in diameter) with a blunt end was pressed against the plantar surface of the paw and a pressure gradient was applied (0 – 50 g) over the course of 20 s. The force at which the rat moved its paw was used as the dependent measure of mechanical sensitivity. Both hind paws were tested on each rat 3 times and the average of the 3 tests was used as the data point for that time period. A rest period of at least 2 minutes was observed before the animals were retested to prevent sensitization.

### Immunoprecipitation and Western Blots

The rats were euthanized at the indicated times with CO_2 _and the spinal cords were removed by pressure ejection with 5 mls ice cold phosphate buffered saline (pH 7.4). The L2 to L5 region of the cord was isolated and divided in half lengthwise. The side ipsilateral to the paw injection was retained for the experiments. The tissue was immediately sonicated and boiled for 10 minutes in denaturing buffer (10 mls/g tissue) containing 1% SDS, 10 mM Tris pH 7.4 and 0.4 mM sodium ortho-vanadate. The solution was then centrifuged at 25,000 g for 30 minutes and the supernatant collected. A sample of the supernatant containing 500 μg of protein was diluted to 500 μl with deionized water and mixed with 500 μl 2 × immunoprecipitation buffer containing 2% triton X-100, 300 mM NaCl, 20 mM Tris pH 7.4, 2 mM EDTA, 2 mM EGTA, 0.4 mM sodium ortho-vanadate, 0.4 mM PMSF and 1% NP-40. The anti-NR1 or anti-NR2 antibodies (2–5 μg) were added to the solution and incubated over night at 4°C. The proteins were then precipitated with agarose beads coupled to the appropriate anti-IgG antibody (Sigma, St. Louis, MO). The beads were isolated and washed in immunoprecipitation buffer. The beads were then suspended in 30 μl immunoprecipitation buffer and diluted with 30 μl 2 × Laemmli buffer (BioRad, Hercules, CA) and boiled for 10 minutes. The samples were run on 3 – 20% SDS-PAGE gels and semi-dry transferred to PVDF membranes. Phospho-serine, -threonine and -tyrosine were then examined using the appropriate phospho-selective anti-bodies (Sigma, St. Louis, MO). The bands were viewed using HRP chemiluminescence and film. Band density was determined using Scion Image (Scion corporation, Gaithersburg, MD). To verify that the immunoprecipitations were quantitative samples of the supernatant following the removal of the beads were run on western blots and probed with the appropriate anti-NR1 or anti-NR2 antibodies. If the precipitated protein was found in the supernatant the precipitation procedure was repeated.

To analyze NR1 and NR2 protein a sample of the denaturing buffer containing 20 μg of protein was mixed with an equal volume of 2 × Laemmli buffer, boiled (10 minutes) and run as described above. The membranes were then probed with the appropriate antibodies. Lane loading was verified with anti-actin antibodies. The density of the actin bands was determined. If the density of any band in the gel diverged more than one standard deviation from the mean of all the bands the gel was discarded and the experiment repeated. All statistics were performed on the raw band density, but were presented graphically as data normalized to the non-inflamed control data to enhance clarity.

Rabbit antibodies to the exon 5 region (N1), exon 21 region (C1), exon 22 C-terminus (C2) and exon 22 minus C-terminus (C2') cassettes [18] of the NR1 subunit were raised at the National Institute for Dental and Craniofacial Research, National Institutes of Health using the following peptide sequences (N1) SKKRNYENLDQLSYDNKRGPC, (C1) DRKSGRAEPDPKKKATFRAC, (C2) PRRAIEREEGQLQLC and (C2') QYHPTDITGPLNLSDPS. The antibodies were affinity purified and tested for specificity using COS-7 cells transfected with specific NR1 splice variants and western blots (Figure 1). The secondary HRP coupled antibodies were purchased from Sigma (ST. Louis, MO). The remainder of the antibodies were purchased from Chemicon International (Temecula, CA), BD Biosciences (San Jose, CA) or Santa Cruz Biotechnology (Santa Cruz, CA).

### Data Analysis

Western blot band density was measured using Scion Image (Scion Corp., Frederick, MD). All statistical analyses were conducted on the raw data. Statistical analysis consisted of ANOVA followed by Dunnett's tests or Deming linear regression using Prism statistical software (Graphpad Software inc., San Diego, CA). Statistical significance was assigned to p ≤ 0.05.

## Competing interests

The author(s) declare that they have no competing interests.

## Authors' contributions

RMC designed the study, ran the behavioral experiments and prepared the manuscript. FMP performed immunoprecipitations and western blots. AYD performed immunoprecipitations and western blots. MJI raised the NR1 splice variant selective antibodies.

## References

[B1] Vierck CJ, Cannon RL, Fry G, Maixner W, Whitsel BL (1997). Characteristics of temporal summation of second pain sensations elicited by brief contact of glabrous skin by a preheated thermode. Journal of Neurophysiology.

[B2] Urch CE, Rahman W, Dickenson AH (2001). Electrophysiological studies on the role of the NMDA receptor in nociception in the developing rat spinal cord. Developmental Brain Research.

[B3] Woolf CJ, Thompson SWN (1991). The Induction and Maintenance of Central Sensitization Is Dependent on N-Methyl-D-Aspartic Acid Receptor Activation - Implications for the Treatment of Postinjury Pain Hypersensitivity States. Pain.

[B4] Ren K, Dubner R (1999). Central nervous system plasticity and persistent pain. Journal of Orofacial Pain.

[B5] Ren K, Dubner R (1993). Nmda Receptor Antagonists Attenuate Mechanical Hyperalgesia in Rats with Unilateral Inflammation of the Hindpaw. Neuroscience Letters.

[B6] Ren K, Hylden JLK, Williams GM, Ruda MA, Dubner R (1992). The Effects of A Noncompetitive Nmda Receptor Antagonist, Mk-801, on Behavioral Hyperalgesia and Dorsal Horn Neuronal-Activity in Rats with Unilateral Inflammation. Pain.

[B7] Ren K, Williams GM, Hylden JLK, Ruda MA, Dubner R (1992). The Intrathecal Administration of Excitatory Amino-Acid Receptor Antagonists Selectively Attenuated Carrageenan-Induced Behavioral Hyperalgesia in Rats. European Journal of Pharmacology.

[B8] Ji RR, Woolf CJ (2001). Neuronal plasticity and signal transduction in nociceptive neurons: implications for the initiation and maintenance of pathological pain. Neurobiol Dis.

[B9] Woolf CJ, Thompson SW, King AE (1988). Prolonged primary afferent induced alterations in dorsal horn neurones, an intracellular analysis in vivo and in vitro. J Physiol (Paris).

[B10] Woolf CJ (1996). Windup and central sensitization are not equivalent. Pain.

[B11] Zou XJ, Lin Q, Willis WD (2000). Enhanced phosphorylation of NMDA receptor 1 subunits in spinal cord dorsal horn and spinothalamic tract neurons after intradermal injection of capsaicin in rats. Journal of Neuroscience.

[B12] Crump FT, Dillman KS, Craig AM (2001). cAMP-dependent protein kinase mediates activity-regulated synaptic targeting of NMDA receptors. Journal of Neuroscience.

[B13] Zou X, Lin Q, Willis WD (2002). Role of protein kinase a in phosphorylation of NMDA receptor 1 subunits in dorsal horn and spinothalamic tract neurons after intradermal injection of capsaicin in rats. Neuroscience.

[B14] Brenner GJ, Ji RR, Shaffer S, Woolf CJ (2004). Peripheral noxious stimulation induces phosphorylation of the NMDA receptor NR1 subunit at the PKC-dependent site, serine-896, in spinal cord dorsal horn neurons. Eur J Neurosci.

[B15] Scott DB, Blanpied TA, Ehlers MD (2003). Coordinated PKA and PKC phosphorylation suppresses RXR-mediated ER retention and regulates the surface delivery of NMDA receptors. Neuropharmacology.

[B16] Caudle RM, Perez FM, King C, Yu CG, Yezierski RP (2003). N-methyl-D-aspartate receptor subunit expression and phosphorylation following excitotoxic spinal cord injury in rats. Neurosci Lett.

[B17] Guo W, Zou SP, Guan Y, Ikeda T, Tal M, Dubner R, Ren K (2002). Tyrosine phosphorylation of the NR2B subunit of the NMDA receptor in the spinal cord during the development and maintenance of inflammatory hyperalgesia. Journal of Neuroscience.

[B18] Zukin RS, Bennett MV (1995). Alternatively spliced isoforms of the NMDARI receptor subunit. Trends Neurosci.

[B19] Petrenko AB, Yamakura T, Baba H, Shimoji K (2003). The role of N-methyl-D-aspartate (NMDA) receptors in pain: a review. Anesth Analg.

[B20] Riedel W, Neeck G (2001). Nociception, pain, and antinociception: current concepts. Zeitschrift fur Rheumatologie.

[B21] Willis WD (2001). Role of neurotransmitters in sensitization of pain responses. Role of Neural Plasticity in Chemical Intolerance.

[B22] Prybylowski KL, Grossman SD, Wrathall JR, Wolfe BB (2001). Expression of splice variants of the NR1 subunit of the N-methyl-D-aspartate receptor in the normal and injured rat spinal cord. Journal of Neurochemistry.

[B23] Gaunitz C, Schuttler A, Gillen C, Allgaier C (2002). Formalin-induced changes of NMDA receptor subunit expression in the spinal cord of the rat. Amino Acids.

[B24] Nagy GG, Watanabe M, Fukaya M, Todd AJ (2004). Synaptic distribution of the NR1, NR2A and NR2B subunits of the N-methyl-d-aspartate receptor in the rat lumbar spinal cord revealed with an antigen-unmasking technique. Eur J Neurosci.

[B25] Kovacs G, Kocsis P, Tarnawa I, Horvath C, Szombathelyi Z, Farkas S (2004). NR2B containing NMDA receptor dependent windup of single spinal neurons. Neuropharmacology.

[B26] Harris J, Joules C, Stanley C, Thomas P, Clarke RW (2004). Glutamate and tachykinin receptors in central sensitization of withdrawal reflexes in the decerebrated rabbit. Exp Physiol.

[B27] Tan PH, Yang LC, Shih HC, Lan KC, Cheng JT (2005). Gene knockdown with intrathecal siRNA of NMDA receptor NR2B subunit reduces formalin-induced nociception in the rat. Gene Ther.

[B28] Parsons CG, Danysz W, Quack G (1998). Glutamate in CNS disorders as a target for drug development: an update. Drug News Perspect.

[B29] Chazot PL (2004). The NMDA receptor NR2B subunit: a valid therapeutic target for multiple CNS pathologies. Curr Med Chem.

[B30] Smith PF (2003). Therapeutic N-methyl-D-aspartate receptor antagonists: will reality meet expectation?. Curr Opin Investig Drugs.

[B31] LoGrasso P, McKelvy J (2003). Advances in pain therapeutics. Curr Opin Chem Biol.

[B32] Nakazato E, Kato A, Watanabe S (2005). Brain but not spinal NR2B receptor is responsible for the anti-allodynic effect of an NR2B subunit-selective antagonist CP-101,606 in a rat chronic constriction injury model. Pharmacology.

[B33] De Vry J, Kuhl E, Franken-Kunkel P, Eckel G (2004). Pharmacological characterization of the chronic constriction injury model of neuropathic pain. Eur J Pharmacol.

[B34] Minami T, Matsumura S, Okuda-Ashitaka E, Shimamoto K, Sakimura K, Mishina M, Mori H, Ito S (2001). Characterization of the glutamatergic system for induction and maintenance of allodynia. Brain Res.

[B35] Hawkins LM, Prybylowski K, Chang K, Moussan C, Stephenson FA, Wenthold RJ (2004). Export from the endoplasmic reticulum of assembled N-methyl-d-aspartic acid receptors is controlled by a motif in the c terminus of the NR2 subunit. J Biol Chem.

[B36] Prybylowski K, Fu Z, Losi G, Hawkins LM, Luo J, Chang K, Wenthold RJ, Vicini S (2002). Relationship between availability of NMDA receptor subunits and their expression at the synapse. J Neurosci.

[B37] Hargreaves K, Dubner R, Brown F, Flores C, Joris J (1988). A new and sensitive method for measuring thermal nociception in cutaneous hyperalgesia. Pain.

